# Sodium-Glucose Linked Cotransporter-2 Inhibition Does Not Attenuate Disease Progression in the Rat Remnant Kidney Model of Chronic Kidney Disease

**DOI:** 10.1371/journal.pone.0144640

**Published:** 2016-01-07

**Authors:** Yanling Zhang, Kerri Thai, David M. Kepecs, Richard E. Gilbert

**Affiliations:** Keenan Research Centre for Biomedical Science and Li Ka Shing Knowledge Institute of St. Michael’s Hospital, Toronto, Canada; INSERM, FRANCE

## Abstract

Pharmacological inhibition of the proximal tubular sodium-glucose linked cotransporter-2 (SGLT2) leads to glycosuria in both diabetic and non-diabetic settings. As a consequence of their ability to modulate tubuloglomerular feedback, SGLT2 inhibitors, like agents that block the renin-angiotensin system, reduce intraglomerular pressure and single nephron GFR, potentially affording renoprotection. To examine this further we administered the SGLT2 inhibitor, dapagliflozin, to 5/6 (subtotally) nephrectomised rats, a model of progressive chronic kidney disease (CKD) that like CKD in humans is characterised by single nephron hyperfiltration and intraglomerular hypertension and where angiotensin converting enzyme inhibitors and angiotensin receptor blockers are demonstrably beneficial. When compared with untreated rats, both sham surgery and 5/6 nephrectomised rats that had received dapagliflozin experienced substantial glycosuria. Nephrectomised rats developed hypertension, heavy proteinuria and declining GFR that was unaffected by the administration of dapagliflozin. Similarly, SGLT2 inhibition did not attenuate the extent of glomerulosclerosis, tubulointerstitial fibrosis or overexpression of the profibrotic cytokine, transforming growth factor-ß1 mRNA in the kidneys of 5/6 nephrectomised rats. While not precluding beneficial effects in the diabetic setting, these findings indicate that SGLT2 inhibition does not have renoprotective effects in this classical model of progressive non-diabetic CKD.

## Introduction

The progressive nature of kidney disease, almost regardless of aetiology, suggests common responses to injury that may be likewise amenable to common therapeutic strategies. Consistent with this observation and in conjunction with experimental findings in rodents, Brenner and colleagues formulated a unifying hypothesis to explain the progressive nature of CKD [1]. According to this haemodynamic-based theory, injury-mediated focal nephron loss leads to hypertrophy, hyperfiltration and increased pressure among remaining glomeruli. These responses, serving to increase GFR in remaining nephrons, whilst initially beneficial are ultimately detrimental, increasing glomerular work and causing barotrauma.

Proof-of-concept studies that seem to confirm the hemodynamic hypothesis of CKD progression were founded, in part, on studies in the remnant kidney model where the increase in single nephron glomerular filtration rate (↑SNGFR) among remaining glomeruli arises from a proportionally greater increase in efferent versus afferent arteriolar resistance [2]. The resultant increase in intraglomerular pressure (P_GC_), leads in turn, to intraglomerular hypertension whereby barotrauma induces glomerulosclerosis, proteinuria and declining filtration. Although not associated with nephron loss, a similar scenario is thought to apply to diabetes where glomerular hyperfiltration occurs as a consequence of relative afferent arteriolar dilatation in individuals with recent onset diabetes [3] and rats with streptozotocin-induced diabetes [4]. As a corollary, strategies that reduce intraglomerular pressure should be renoprotective in both non-diabetic and diabetic kidney disease. Indeed, blocking angiotensin II’s predilection for preferentially increasing efferent arteriole tone and thereby elevating intraglomerular pressure [5] has placed angiotensin converting enzyme inhibitors and angiotensin receptor blockers at the cornerstone of renal medicine in most CKD settings.

In addition to lessening angiotensin II-dependent efferent arteriolar constriction, intraglomerular pressure may also be reduced by increasing afferent arteriolar tone. Indeed, these latter changes can be induced by modulating tubuloglomerular feedback (TGF) function where the physiological response to excessive delivery of NaCl to the distal nephron includes afferent arteriolar constriction and consequent reductions in both SNGFR and P_GC_ [6]. Accordingly, reducing proximal tubular Na^+^ reabsorption by blocking the activity of sodium-glucose cotransporter 2 (SGLT2), leads to a reduction in GFR in humans with diabetes-associated hyperfiltration [7] and in both total and single nephron GFR in rodents with diabetes [8]. These data have led to the suggestion that SGLT2 inhibitors, by reducing hyperfiltration and by also possibly lowering P_GC_ may, akin to agents that block the RAS, be renoprotective [9–11].

While studies with SGLT2 inhibitors have been conducted almost entirely in the diabetes, this drug class lowers tubular glucose reabsorption sufficiently to induce glycosuria in both animal models and human subjects without hyperglycaemia [12–16]. As such, by modulating TGF, SGLT2 inhibition’s effects on SNGFR and P_GC_ should also apply to the non-diabetic setting, the other differences between diabetic and non-diabetic kidney disease not withstanding. Accordingly, like the original experiments by Brenner and colleagues with ACE inhibition in both the remnant kidney and diabetic rat models [17, 18], we postulated that SGLT2 inhibition might be similarly renoprotective in the non-diabetic setting.

## Materials and Methods

### Animals

Fifty-three male Sprague-Dawley rats (Charles River, Montreal, Quebec), aged 10 weeks were randomly assigned to undergo subtotal nephrectomy or sham surgery. Subtotal (5/6) nephrectomy (SNX) was performed in a one-step procedure, as previously described [19] whereby animals were under 2.5% isoflurane anaesthesia the right kidney was excised and infarction of approximately two thirds of the left kidney was achieved via selective ligation of 2 out of the 3 or 4 branches of the renal artery. Sham surgery consisted of laparotomy and manipulation of both kidneys before wound closure. Rats were maintained at the St. Michael’s Hospital Animal Research Vivarium in a temperature-controlled (22°C) room with *ad libitum* access to commercial standard rat chow. All animal studies were approved by the St. Michael’s Hospital Animal Ethics Committee in accordance with the Guide for the Care and Use of Laboratory Animals (NIH Publication No. 85–23, revised 1996).

One week after surgery, sham and nephrectomised animals were randomly assigned to receive dapagliflozin (0.5 mg/kg, twice/day, Shanghai Sun-shine chemical Technology Co., Ltd.) or vehicle (5% 1-methyl-2-pyrrolidinone, 20% polyethylene glycol, and 20 mmol/l sodium diphosphate) by gavage and followed for a total of 12 weeks.

### Biochemistry and function

Body weight was measured monthly. Systolic blood pressure was measured every four weeks in conscious rats using an occlusive tail-cuff plethysmograph attached to a pneumatic pulse transducer (Powerlab, AD Instruments, Colorado Springs, CO), as previously described [20]. Similarly, every four weeks, animals were housed in individual metabolic cages for 24 hours in order to determine their daily water intake, urine output and urine protein excretion using the benzethonium chloride method and urinary glucose excretion by glucose oxidase technique (Eton Biosciences, San Diego, CA).

Glomerular filtration rate (GFR) was assessed at 8 weeks and 12 weeks post surgery, using a modified FITC-inulin plasma clearance assay, as previously reported [21]. In brief, FITC-inulin (3.74 μL/g body weight) was injected via the tail vein and venous blood sampled at various time points subsequently. Fluorescence was detected using a Fluoroscan Ascent FL machine (Thermo Scientific, Rockford, IL) with 485 nm excitation and 538 nm emission settings and GFR was calculated using the following two phase, exponential decay curve with non-linear regression statistics, as previously described [21], whereby GFR = I/(A/α + B/β), where I is the amount of FITC-inulin injected, A and B are the y-intercept values for the two decay rates, and α and β are the decay constants for the distribution and elimination phases. As a further marker of GFR, creatinine was measured in plasma and urine by autoanalyzer (Beckman Instruments, Palo Alto, CA), as previously reported [22], and the creatinine clearance calculated.

### Tissue preparation and histochemistry

At the end of the study, 12 weeks after SNX surgery, rats were anesthetized with inhaled isoflurane 2.5%. The left renal artery was clamped and the remnant kidney removed, decapsulated, and sliced transversely before immersion fixation in 10% neutral buffered formalin (NBF), embedding in cryostat matrix (Tissue-Tek, Sakura, Kobe, Japan), or flash frozen in liquid nitrogen. Formalin-fixed tissues were routinely processed, embedded in paraffin, and sectioned before staining with Periodic acid-Schiff (PAS) or Masson’s trichrome, as previously reported [23].

### Glomerulosclerosis and interstitial fibrosis

Microscopy was performed by observers who were masked to the study group from which the kidney sections were derived. Glomerulosclerosis was assessed in 4 μm thick sections using a semi-quantitative technique, as previously described [19]. In brief, the extent of sclerosis was graded subjectively in each glomerulus on a scale of 0–4: Grade 0, normal; Grade 1, sclerotic area up to 25% (minimal); Grade 2, sclerotic area 25–50% (moderate); Grade 3, sclerotic area 50–75% (moderate to severe) and Grade 4, sclerotic area 75–100% (severe). The glomerulosclerotic index (GSI) was then calculated using the formula: GSI = (1 × n_1_ + 2 × n_2_ + 3 × n_3_ + 4 × n_4_)/(n_0_ + n_1_ + n_2_ + n_3_ + n_4_), where n_x_ is the number of glomeruli in each grade of glomerulosclerosis. The extent of interstitial fibrosis was quantified in 4 μm kidney sections stained with Masson’s trichrome. Stained sections were scanned with the Aperio Ultra-Resolution Digital Scanning System (Aperio Technologies Inc., Vista, CA), and images were analyzed with Aperio ImageScope software. An area of blue in cortex of the kidney was selected for its colour range and the proportional area of the selected colour range was then quantified, based on the method adapted from Lehr et al. [24, 25]. Ten randomly selected non-overlapping 10x fields for each animal was analysed in a masked fashion. Data were expressed as percentage change per area.

### Real-Time quantitative RT-PCR

Quantitative real time PCR was performed using SYBR green with the abundance of transcript expressed relative to that of the housekeeping gene Rpl13a. In brief, frozen kidney tissue that had been stored at -80°C, was homogenized (Polytron, Kinematica Gmbh, Littau, Switzerland) and total RNA was isolated using TRIzol reagent (Life Technologies, Grand Island, NY). Total RNA (4μg) was treated with RQ1 DNAse (1U/μl) (Promega, Madison, WI) to remove genomic DNA. RNA was reverse transcribed with 1μl of random hexamers (2μg/μl) and 8μl of DEPC-treated water and incubated for 5min at 70°C. After cooling on ice, 5μl of 5x AMV reaction buffer, 2.5μl of 10mmol/L dNTP mix, 0.5μl RNase inhibitor (40U/μl) (Roche, Indianapolis, IN), 0.5μl AMV reverse transcriptase (25U/μl) (Roche) and 4.5μl DEPC water were added. The reaction mixture was incubated for 60 minutes and the cDNA samples were stored at -20°C. Real-time PCR was performed on an ABI Prism 7900HT Fast PCR System (Applied Biosystems, Foster City, CA) using sequence specific primers for TGF-ß1, α1 (IV) collagen, α1 (I) collagen and RPL13a were obtained from Applied Biosystems (Foster City, CA). Experiments were performed in triplicate and data analysis was performed using the Comparative C_T_ method (Sequence Detection Systems 2.0; Applied Biosystems).

### Immunohistochemistry

In addition to examining TGF-ß1 mRNA we also assessed activation of the TGF-ß signalling pathway by quantifying nuclear expression of phosphorylated Smad2 using a rabbit antiphospho-Smad2 antibody (Cell Signalling Technology, Boston, MA, USA) that detects endogenous Smad2 only when dually phosphorylated at Ser465 and Ser467. In brief and as previously described [26, 27], sections were immunostained according to the manufacturer’s instructions and the number of immunolabelled nuclei quantified in 5 random non-overlapping fields per kidney by an observer who was masked to the identity of the experimental group.

Macrophages were identified by the monoclonal rat macrophage marker (ED-1; Serotec, Raleigh, NC), as previously reported [28] and quantified as described above for phospho-Smad2. Type I collagen was examined immunohistochemically using an anti-type I collagen antibody (Southern Biotechnology Associates Inc, Birmingham, Ala), as previously reported [29] with the extent of its immunolabelling quantified as the proportional area in 5 random non-overlapping fields per kidney.

### Statistics

Data are expressed as means ± SEM. Between group differences were analysed by one way ANOVA with Tukey’s post hoc test for multiple comparisons. Survival analysis was conducted according to the method of Kaplan and Meier. All statistics were performed using GraphPad Prism 5 for Mac OS X (GraphPad Software Inc., San Diego, CA). A p value of <0.05 was considered statistically significant.

## Results

### Animal data

Dapagliflozin, commenced one week after sham surgery or SNX induced substantial glycosuria contrasting its near absence in vehicle-treated animals (Table 1). Urine output was higher in dapagliflozin-treated rats when compared with their untreated counterparts. Body weight was lower in SNX rats compared with shams.

**Table 1:** Animal characteristic and kidney function parameters.

	sham + vehicle	sham + dapa	SNX + vehicle	SNX + dapa
Body weight (g)	707.5 ± 10.7	659 ± 21.9	631.1 ± 25.8	568.5 ± 21.3*
Urine output (ml/day)	33.1 ± 2.4	64 ± 4.2*	43 ± 2.4	71.9 ± 3.5*†
Urine glucose (mg/day)	13.9 ± 8.6	1569.7 ± 107.3*	2.3 ± 0.5	1061.4 ± 267.9*†

* p < 0.05 vs. sham + vehicle group;

^†^ p < 0.05 vs. SNX + vehicle group.

### Kidney function and mortality

**Fig 1:**
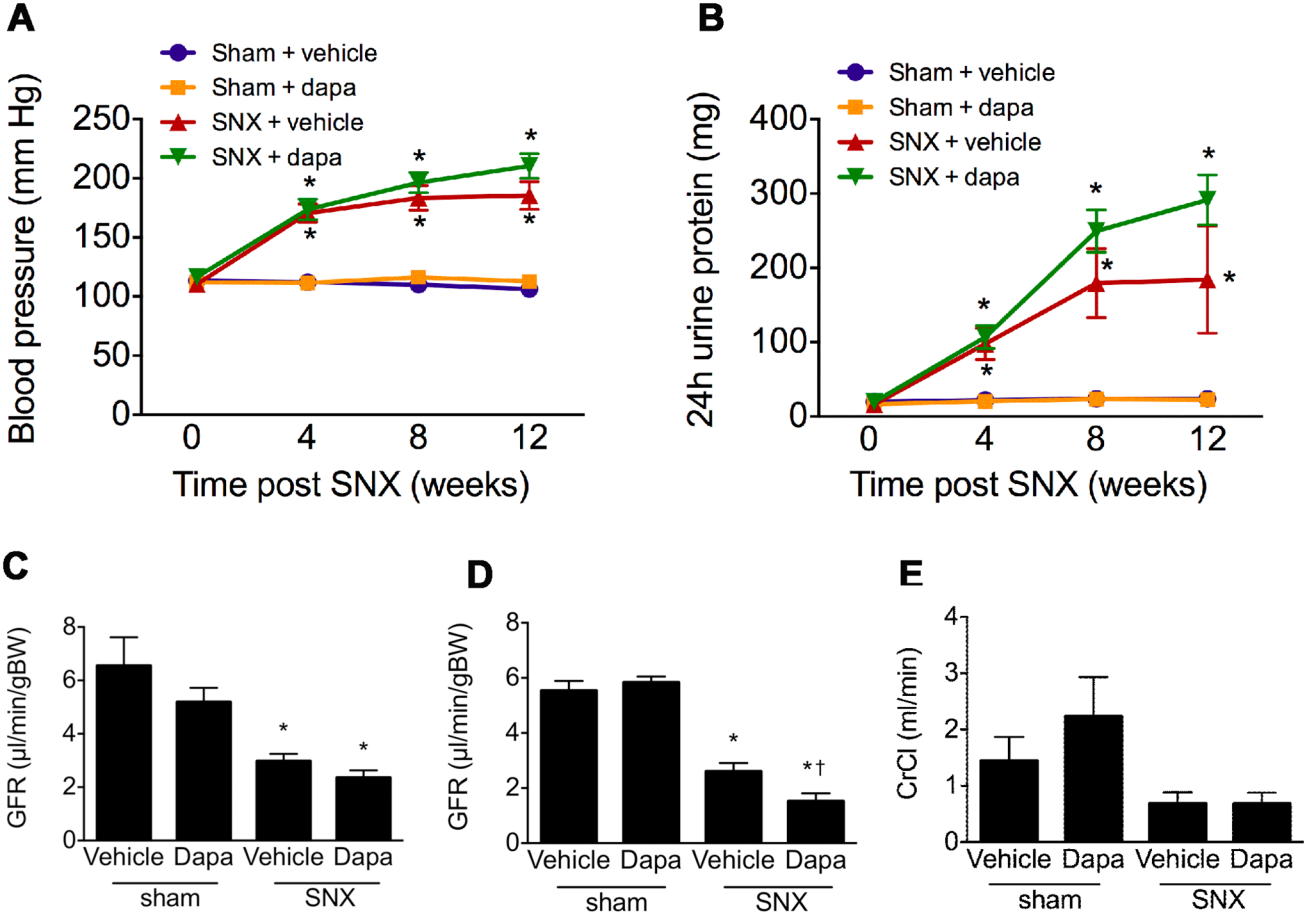
Kidney function. Blood pressure (A) and proteinuria (B) at 4, 8 and 12 weeks and GFR (C-E) as assessed by FITC inulin clearance at 8 weeks (C) and by both FITC inulin (D) and creatinine clearance (CrCl, E) at 12 weeks following subtotal nephrectomy or sham surgery, treated with either vehicle or dapagliflozin. When compared with sham nephrectomised rats, animals that had undergone subtotal nephrectomy developed hypertension, heavy proteinuria and reduced GFR. Subtotally nephrectomised animals that had received dapaglaflozin had similar blood pressure and proteinuria when compared with those that had received vehicle with lower GFR at 12 but not 8 weeks post surgery when assessed by FITC inulin but not by creatinine clearance. * p < 0.05 vs. sham-operated animals, † p < 0.05 vs. vehicle-treated subtotally nephrectomised rats.

**Fig 2:**
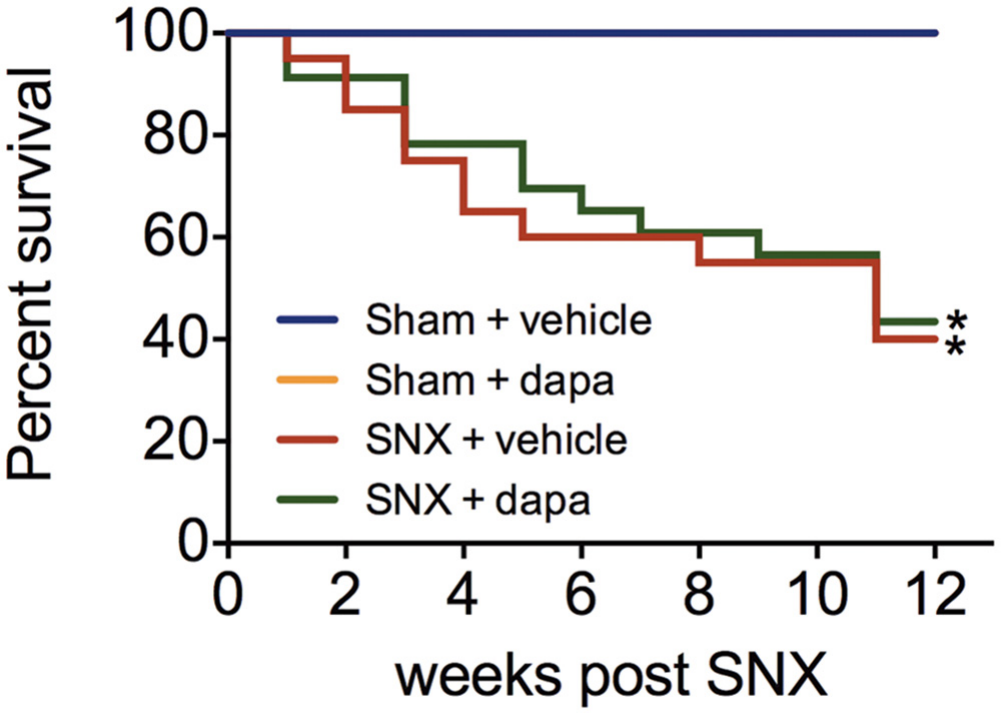
Animal survival. Kaplan-Meier curves show reduced animal survival in SNX rats when compared with animals that had undergone sham surgery. No effect of dapagliflozin on mortality was noted. * p < 0.05 vs. sham-operated animals.

When compared with animals that had undergone sham surgery, subtotally nephrectomised rats developed substantial proteinuria and hypertension (Fig 1A and 1B). No difference in either blood pressure or the magnitude of proteinuria was evident between animals that had received dapagliflozin or vehicle. At eight weeks post surgery, GFR, as measured by FITC-inulin clearance was markedly impaired in rats that had undergone subtotal nephrectomy and to a similar extent in dapagliflozin and vehicle-treated groups (Fig 1C). At 12 weeks, GFR was slightly lower in SNX rats that had received dapagliflozin when compared with those that had received vehicle (Fig 1D) when measured by FITC-inulin clearance. These differences were, however, no longer evident when GFR was assessed by creatinine clearance. Mortality was increased in subtotal nephrectomised rats but was similar in those that received dapagliflozin or vehicle (Fig 2).

### Histopathology

**Fig 3:**
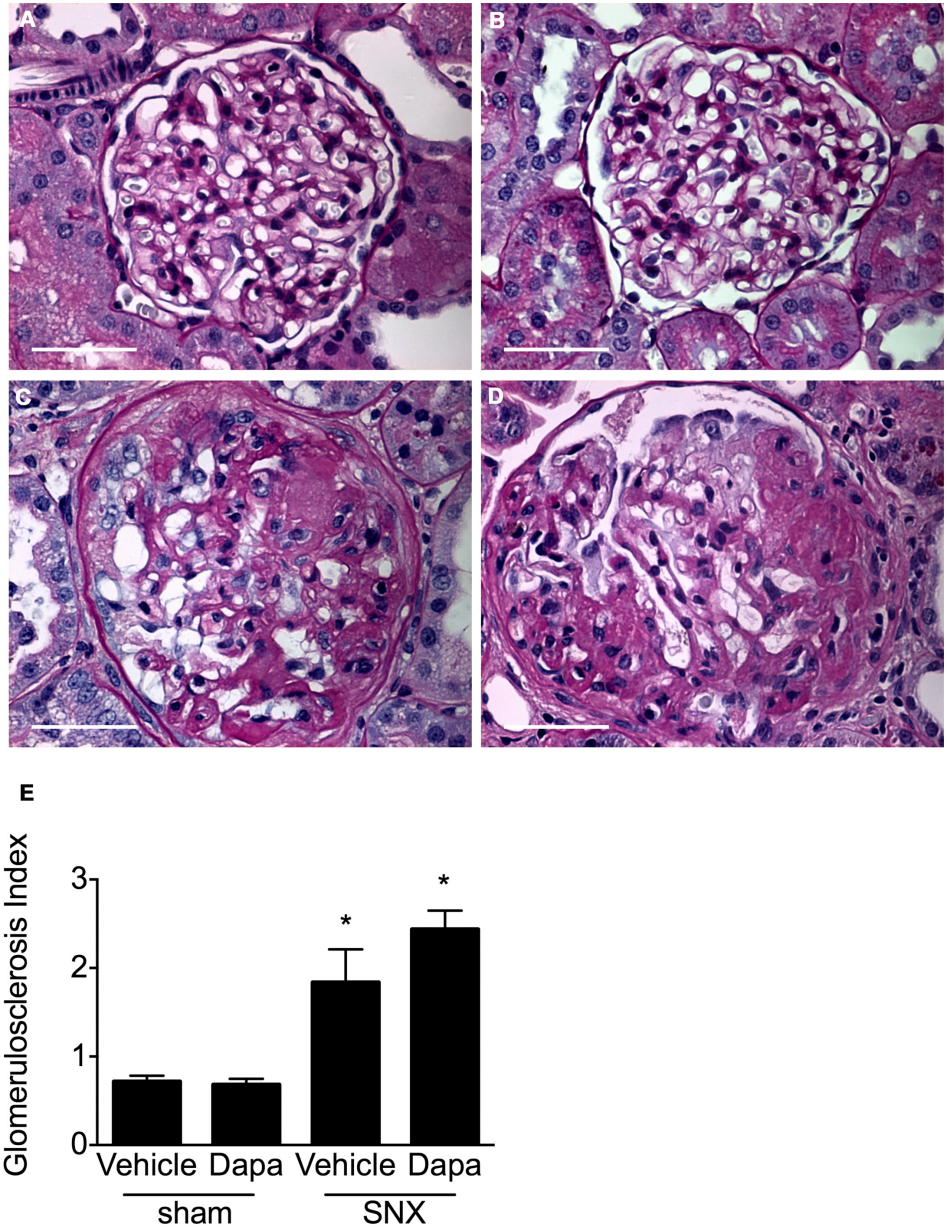
Glomerulosclerosis. Representative glomerular images (A-D) and with quantitative analysis (E) of periodic acid-Schiff stained kidney sections. When compared with animals that had undergone sham surgery (A, B), kidney sections from SNX rats (C, D) show substantial glomerulosclerosis that was similar in vehicle (C) and dapagliflozin-treated rats (D). Original magnification x 400. Scale bars: 50 μm. * p < 0.05 vs. sham-operated animals.

**Fig 4:**
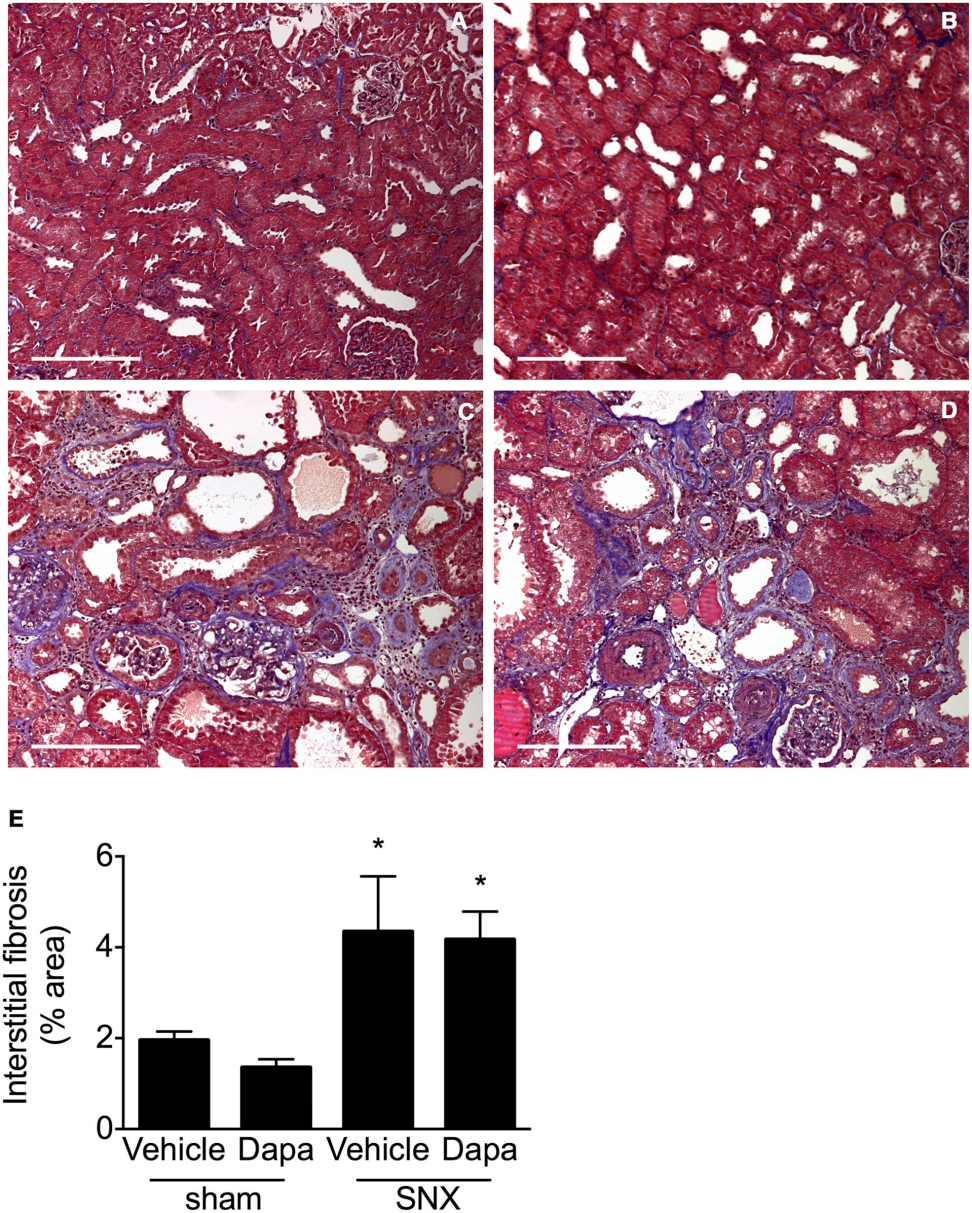
Interstitial fibrosis. Representative cortical tubulointerstitial images (A-D) and with quantitative analysis (E) of Masson’s trichrome-stained kidney sections. When compared with animals that had undergone sham surgery (A, B), kidney sections from SNX rats (C, D) show substantial interstitial fibrosis (blue) that was similar in vehicle (C) and dapagliflozin-treated rats (D). Original magnification x 100. Scale bar: 200 μm. * p < 0.05 vs. sham-operated animals.

By 12 weeks post surgery, examination of PAS-stained kidney sections from subtotal nephrectomised rats revealed substantial and widespread glomerulosclerosis (Fig 3). The extent of the glomerulosclerosis was unaffected by dapagliflozin where the extent of injury was similar to that found in vehicle-treated rats. Marked interstitial fibrosis was also evident in subtotal nephrectomised animals, the extent of which was similar in treated and untreated rats (Fig 4). In sharp contrast, animals that had undergone sham surgery showed no evidence of fibrosis in either their glomeruli or tubulointerstitium, regardless of whether they had been randomised to receive vehicle or dapagliflozin.

### Gene expression

**Fig 5:**
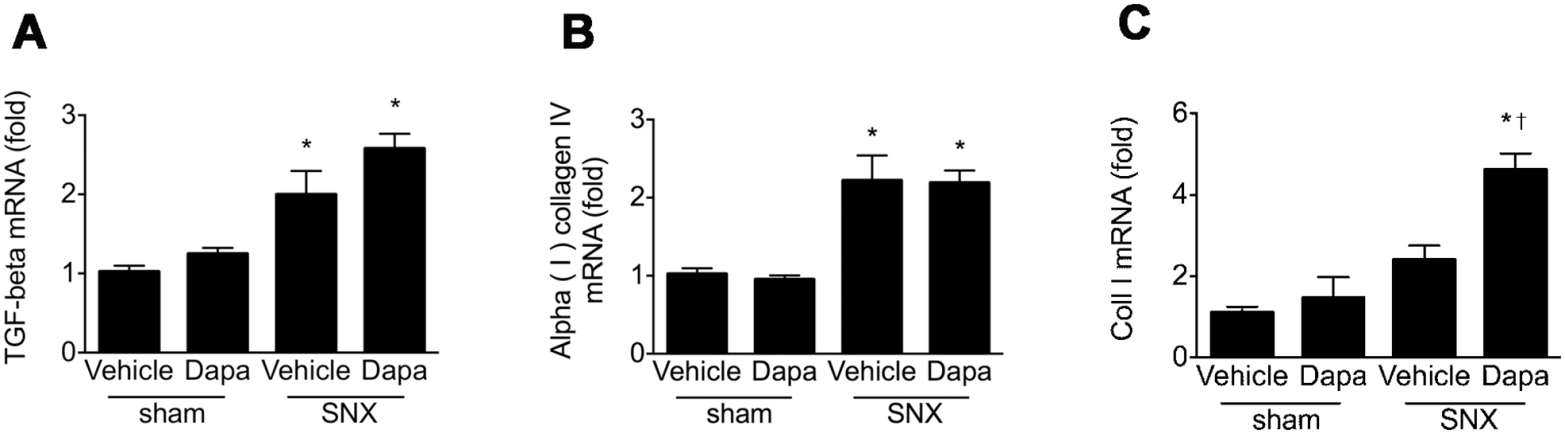
Gene expression. Kidney gene expression of transforming growth factor-ß1, α(I) IV and α(I) I collagen. mRNA was expressed relative to that of RPL13a. The ratio, so-derived was then expressed relative to vehicle-treated rats that had undergone sham surgery that was arbitrarily set at 1. SNX was associated with overexpression of both TGF-ß and α(I) IV collagen that for α(I) I collagen was greater in SNX animals that received dapagliflozin. * p < 0.01 versus sham nephrectomy.

Overexpression of transforming growth factor-ß1 has been repeatedly implicated in the pathogenesis of kidney fibrosis. As expected, TGF-ß1 mRNA was markedly and similarly increased in the kidneys of animals that had undergone subtotal nephrectomy whether they randomised to receive vehicle or dapagliflozin when compared with sham surgery animals. In parallel with these changes in TGF-ß1 gene expression, type IV collagen mRNA was also increased in the kidneys of SNX rats in both dapagliflozin and vehicle-treated groups (Fig 5). Collagen I mRNA was similar in all groups with exception of dapagliflozin-treated SNX rats where expression was increased (Fig 5).

### Immunohistochemistry

**Fig 6:**
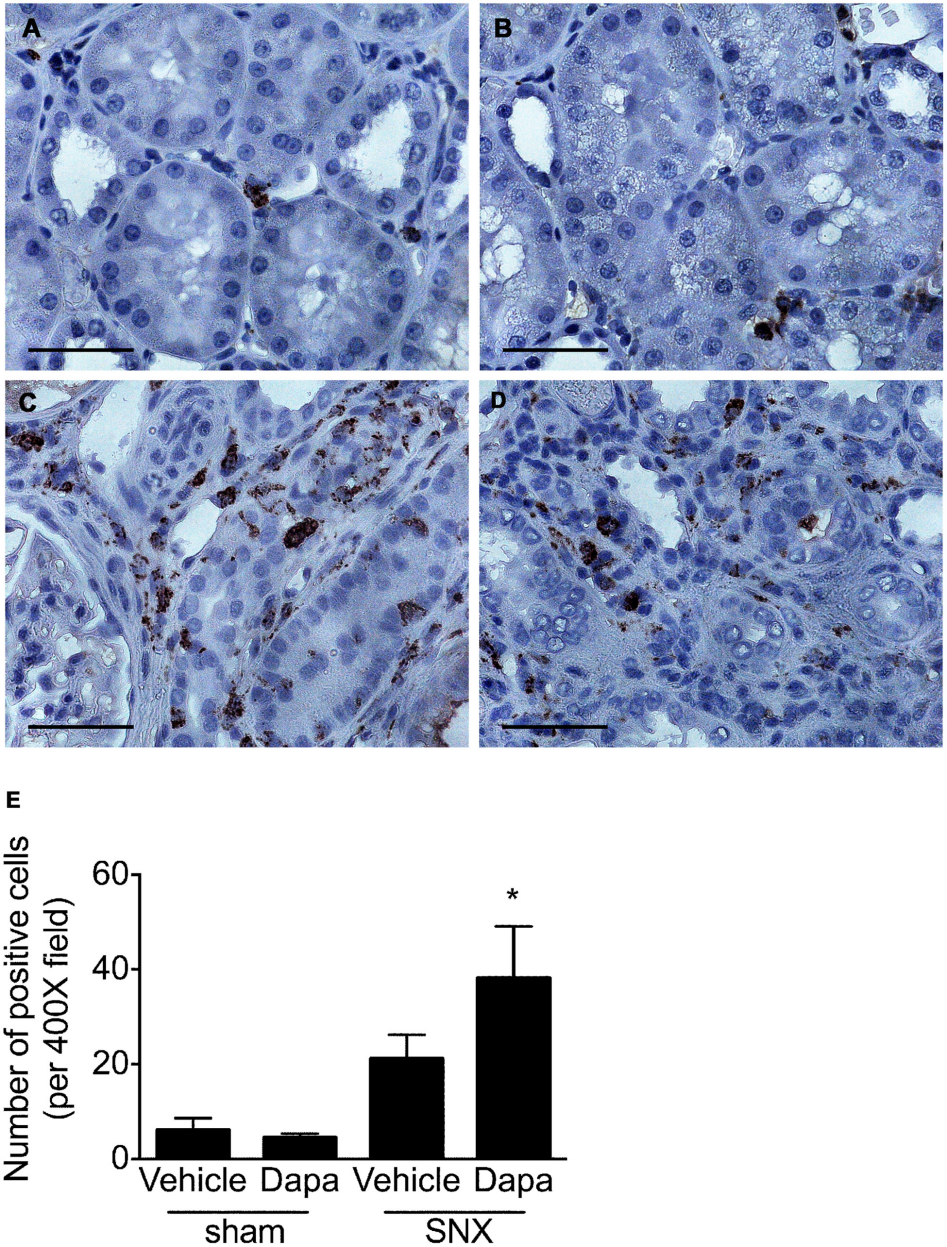
Macrophage infiltration. Kidney sections from SNX rats revealed abundant ED-1 labelled macrophages, predominantly localized to the interstitium that were more common in SNX rats that had received dapagliflozin. Original magnification x 400. Scale bar: 50 μm. * p < 0.05 vs. sham-operated animals.

Macrophages infiltration, as assessed by the number of ED-1 positive cells, was increased in SNX rats but was unaffected by dapagliflozin (Fig 6). These changes were more marked in dapagliflozin-treated SNX animals where the increased abundance reached statistical significance.

**Fig 7:**
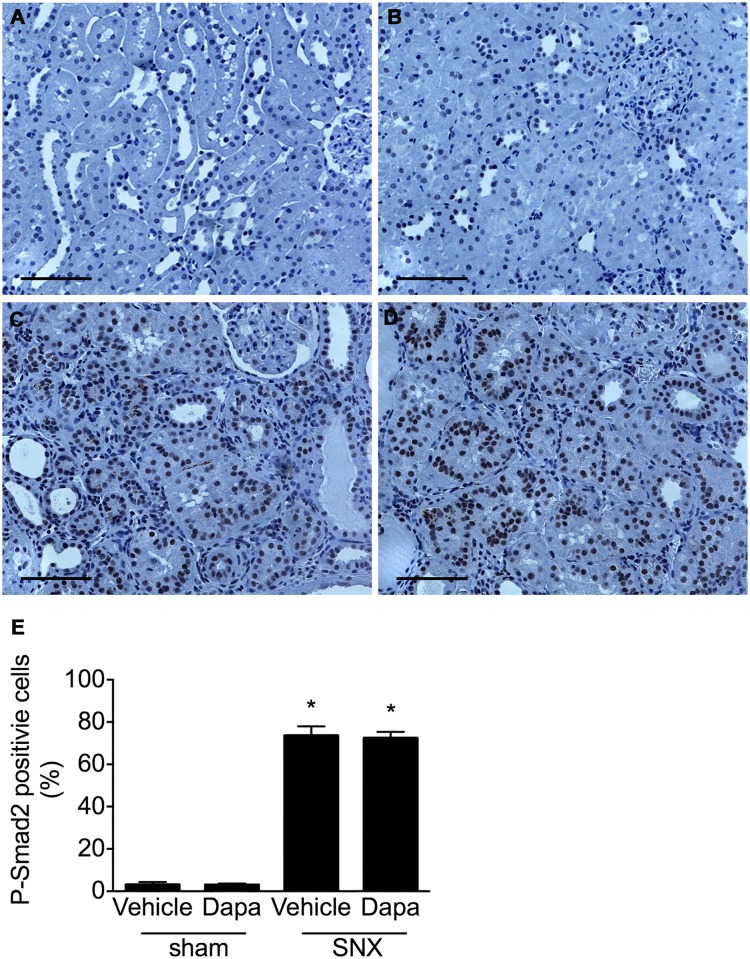
Phosphorylated Smad2. Kidney sections from SNX rats showed abundant nuclear staining for phosphorylated Smad2 in both vehicle and dapagliflozin-treated animals contrasting the dearth of immunopositive cells in sham nephrectomised rats. Original magnification x 160. Scale bar: 100 μm. * p < 0.05 vs. sham-operated animals.

When compared with sham nephrectomised rats, the kidneys of those that had undergone SNX showed a dramatic increase in the abundance of nuclei immunolabelled for phospho-Smad2 consistent with the activation of the TGF-ß signalling pathway (Fig 7). No difference in the proportion of labelled nuclei was found between vehicle and dapagliflozin-treated animals.

**Fig 8:**
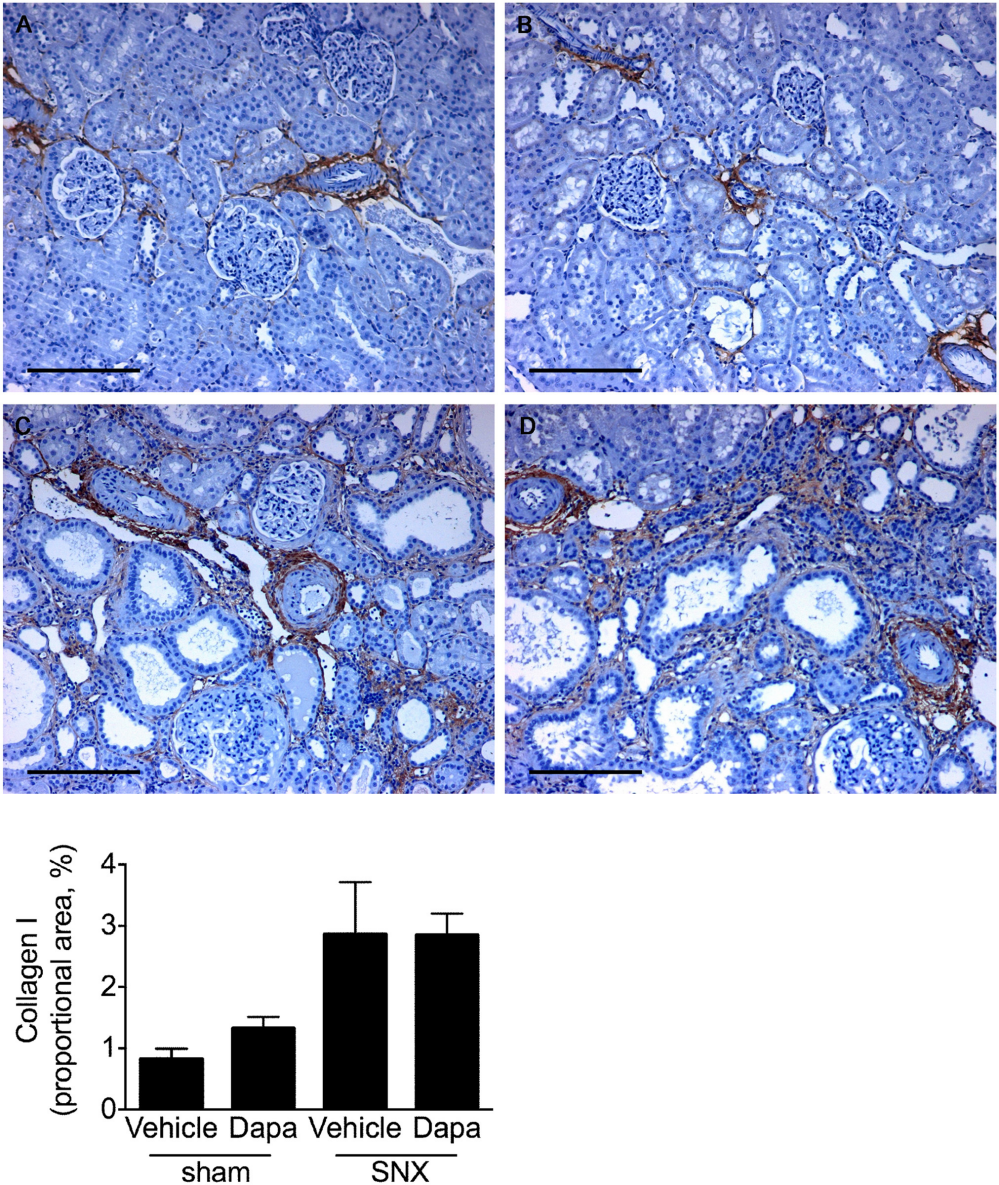
Type I collagen. Representative sections stained with anti-collagen I antibody revealing minimal immunolablelling in sham rats (A, B) while those from SNX animals (C, D) displayed increased deposition surrounding Bowman’s capsule and in bands of interstitial fibrosis. No difference in immunolabelling was evident between those rats that had or had not received dapagliflozin. Original magnification x 100. Scale bar: 200 μm.

Minimal type I collagen was detected in sham animals while in SNX rat kidneys it was noted in areas of marked tubulointerstitial fibrosis and in the arterial adventitia. There was, however, no difference in abundance between those animals that had received dapagliflozin and those that had not (Fig 8)

## Discussion

Reduction in single nephron hyperfiltration and intraglomerular hypertension are fundamental pathophysiological changes that not only underlie the progression of CKD but also provide the rationale for therapeutic strategies to attenuate it. Indeed, blockade of the renin-angiotensin system with ACE inhibitors and ARBs, ostensibly as a consequence of their ability to lower intraglomerular pressure, have been repeatedly shown to reduce the rate of GFR decline in both diabetic and non-diabetic settings [30, 31], placing them along with blood pressure control at the forefront of CKD management. SGLT2 inhibition also leads to an acute reduction in SNGFR in animals [8] and lowering of global GFR in individuals with diabetes [32], akin to the acute reduction in inulin clearance reported with the SGLT1/2 inhibitor, phlorizin, in healthy non-diabetic subjects [33]. SGLT2 inhibition, in the present study, however, did not provide any evidence of renoprotection in the subtotally nephrectomised rat model of non-diabetic CKD.

The hemodynamic hypothesis of CKD progression, posits a vicious cycle whereby hyperfiltration leads to sclerosis that results in a further increase in SNGFR among remaining glomeruli [34]. To examine the ostensibly renoprotective effects of SGLT2 inhibition, we used the subtotally nephrectomized rat, a model of chronic kidney disease, relevant to both diabetic and non-diabetic settings [34] and which develops a decline in GFR, the *sine qua non* of chronic kidney disease. Despite its ability to reduce single nephron and global hyperfiltration in both the animal and human diabetic and non-diabetic settings [7, 8, 32], SGLT2 inhibition in the present study did not affect disease progression in the remnant kidney model. The lack of kidney protection with SGLT2 inhibition in this model contrast the numerous studies showing beneficial effects with agents that block the RAS [17, 19, 35].

A number of very recent studies have addressed the possibility that SGLT2 inhibitors may afford renoprotection in animal models of diabetic nephropathy. A notable caveat for most rodent models of diabetes is that they do not develop a reduced GFR or severe histopathological injury, rather GFR is frequently increased, associated with mild mesangial expansion and modest albuminuria [36, 37]. As such, they mostly resemble early diabetes-associated kidney changes and not the later stages of human diabetic nephropathy where patients present with low GFR, heavy proteinuria and hypertension in conjunction with advanced glomerulosclerosis and marked interstitial fibrosis. Notably, some of the diabetic animal studies that examined the effects of SGLT2 inhibition on GFR, showed a reduction in an elevated GFR rather than a slowing in the progression of CKD [38–41]. Indeed, the reduction in hyperfiltration observed with SGLT2 inhibition in these studies, along with improvements in glycaemic control may well be sufficient to explain the improvements in albuminuria that were also reported.

While RAS blockade and SGLT2 inhibition both reduce hyperfiltration via their hemodynamic actions, angiotensin II, the primary effector molecule of the RAS, also exerts several other effects that contribute to the ability of ACE inhibitors and ARBs to reduce interstitial fibrosis, tubular atrophy and glomerulosclerosis [19, 42, 43]. Notably, acting via its AT_1_ recepetor, angiotensin II potently induces expression of the pro-fibrotic and pro-apoptotic growth factor, transforming growth factor-ß (TGF-ß) in a range of kidney cell types [44, 45]. Concordant with these cell culture studies, TGF-ß overexpression is seen in both the glomerular and tubulointerstitial compartments in the 5/6 nephrectomised and diabetic rats where studies also showed that both ACE inhibitors and ARBs were effective at reducing TGF-ß and disease progression [19, 46]. Consistent with these animal experiments, studies in human diabetic nephropathy showed that the ACE inhibitor, perindopril was able to reduce TGF-ß mRNA in a sequential renal biopsy study [47] and the ARB, losartan, was shown to lower urinary TGF-ß excretion [48]. In contrast to these studies of RAS blockade, SGLT2 inhibition, in the present study, did not affect the expression of TGF-ß or the downstream activation of its canonical Smad signalling pathway. Moreover, expressions of the matrix proteins, collagens I and IV were not improved by the administration of dapagliflozin either. Consistent with these findings SGLT2 inhibition did not affect the extent of glomerulosclerosis, interstitial fibrosis or macrophage infiltration in SNX rats. While dapagliflozin administration led to an increase in collagen I mRNA in SNX rat kidneys, this was not accompanied by a commensurate change in immunostainable collagen that was similarly increased in both treated and untreated SNX rats. The mechanisms underlying the differences between collagen I transcription and protein expression are speculative but could include differences in translation as well as the activities of the collagen I degrading matrix metalloproteinases.

As in the diabetic setting, SGLT2 inhibition has been shown to induce substantial glycosuria in the absence of hyperglycaemia as well [12–16, 49]. As might be expected, rats that were randomised to receive dapagliflozin in the present study developed marked glycosuria and attendant osmotic diuresis with a substantial increase in urinary glucose and comparatively lower body weight. Consistent with GFR as a determinant of SGLT2-induced glycosuria [50], the 24 hour urinary excretion of glucose was less in dapagliflozin treated SNX rats when compared with animals that had only undergone sham surgery. Given the volume contraction that accompanies SGLT2 inhibition [51], there might likely have been a pre-renal component to the reduced GFR seen in dapagliflozin-treated animals that could potentially have masked any improvement in GFR that might have otherwise been detected.

Although single nephron hyperfiltration is a feature of both diabetic and non-diabetic CKD, other aspects of their pathogenesis are substantially different. Indeed, even the genesis of single nephron hyperfiltration is different in the two disease states. In the 5/6 nephrectomized rat, for instance, remaining nephrons hyperfilter to compensate for nephron loss while in diabetes single nephron hyperfiltration may be more a consequence of the proximal tubular growth and generalized kidney enlargement seen early in the disease process [52]. Accordingly, the lack of demonstrable renoprotection in the subtotal nephrectomised rat model, as described in the present study, should not be extrapolated to the diabetic setting where protection from kidney injury has been noted in many [38, 39, 41] but not all studies [53]. As such, the absence of renoprotection in our study should not be viewed as precluding a favourable outcome in kidney disease in individuals with diabetes where a range of non-hemodynamic effects afforded by SGLT2 inhibition may underlie their potential beneficial effects. Indeed, a large multi-centre clinical trial, CREDENCE (The Evaluation of the Effects of Canagliflozin on Renal and Cardiovascular Outcomes in Participants With Diabetic Nephropathy, clinicaltrials.gov identifier: NCT02065791) to address the possible renoprotective properties of SGLT2 inhibition, is currently being conducted in patients with diabetic kidney disease.
